# Laccase-Driven Transformation of High Priority Pesticides Without Redox Mediators: Towards Bioremediation of Contaminated Wastewaters

**DOI:** 10.3389/fbioe.2021.770435

**Published:** 2022-02-11

**Authors:** Vasanth Kumar Vaithyanathan, Vinoth Kumar Vaidyanathan, Hubert Cabana

**Affiliations:** ^1^ University of Sherbrooke Water Research Group, Environmental Engineering Laboratory, Faculty of Engineering, Université de Sherbrooke, Sherbrooke, QC, Canada; ^2^ Integrated Bioprocessing Laboratory, School of Bioengineering, SRM Institute of Science and Technology (SRM IST), Kancheepuram, India

**Keywords:** xenobiotics, biosolids, enzymes, fermentation, pretreament

## Abstract

In this study, *Pleurotus dryinus* was grown on municipal biosolids (BS) as the substrate to produce laccase for the removal of pesticides (fungicides, herbicides, and insecticides) from wastewater. Among the various types of BS tested, sterilized biosolids were the most promising substrate for laccase production by *P. dryinus* with a maximal laccase activity (162.1 ± 21.1 U/g dry substrate), followed by hygenized biosolids (96.7 ± 17.6 U/g dry substrate), unsterilized biosolids (UBS) (31.9 ± 1.2 U/g dry substrate), and alkali-treated biosolids (8.2 ± 0.4 U/g dry substrate). The ultrasound-assisted extraction of this enzyme from fermented UBS was carried out with 0.1 M phosphate buffer at pH 7.0, which increased the enzyme activity of the crude extract by 30%. To test the catalytic potential of the biocatalyst in real matrices, 1 U/ml of recovered crude laccase extract was applied for 24 h for the removal of 29 pesticides (nine fungicides, 10 herbicides, and 10 insecticides) either separately or as a mixture from spiked biologically treated wastewater effluent. When treated with crude enzyme extract, high-priority herbicides metolachlor and atrazine were completely removed, while 93%–97% of the insecticides aldicarb, spinosad, and azinphos-methyl and up to 91% of kresoxim-methyl were removed. Promising results were obtained with BS-derived crude enzyme extract exhibiting improved pesticides removal, which may be due to the mediator effect resulting from the catalytic transformation of other molecules in the cocktail. The results demonstrated a promising integrated bioprocess for the removal of pesticides in wastewater using crude laccase obtained from BS.

## 1 Introduction

Xenobiotics are synthetic chemicals that are structurally foreign to an organism and impose toxic stress due to the organism’s inability to process the compound *via* its metabolic machinery (Ellouze and Sayadi, 2016; Singh, 2017). Unfortunately, for this reason, synthetic pesticides are increasingly in demand in the agrochemical industry to ensure global food security by protecting crop plants against pest attacks (Lushchak et al., 2018). However, indiscriminate use of harmful substances levies a hefty price on the ecological balance, starting from the foundation of a biome to the lives of animals and humans alike. These compounds are primarily toxic due to the complex nature of their molecular makeup, rendering them recalcitrant to simplistic biodegradation, resulting in bio-accumulation at higher levels of the food chain (Parte et al., 2017; Mustafa et al., 2021). Around 2 million tons of pesticides are produced and used worldwide every year. Moreover, a recent study based on a scientific literature review supplemented by the World Health Organization (WHO) mortality database indicates that 385 million cases of unintentional acute pesticide poisoning occur worldwide annually (Sharma et al., 2019; Boedeker et al., 2020). With respect to pesticide use, global statistics indicate that herbicides account for 47.5% of the total pesticide consumption, while insecticides, fungicides, and other pesticides account for 29.5, 17.5, and 5.5% respectively (Sharma et al., 2019).

Every year, approximately 35 million kg of pesticides are used in agricultural fields in Canada and around 56 million kg in India (Sharma et al., 2019). Of these, herbicides are the most prominent pesticide used in Canada, whereas in India, insecticides are the prominent one. A health study conducted from the agriculture-dominated population in India indicated that the blood samples collected tested positive for pesticide residues (Sharma et al., 2015), while another health study indicated that the exposure to pesticides during fetal development could be linked with cognitive deficits in 3-to-4-year-old children and pesticide exposure in agriculture workers could be linked with hematopoietic cancers (Kachuri et al., 2017; Ntantu Nkinsa et al., 2020).

Considering the widespread and indispensable use of pesticides in the agriculture sector, efficient remediation strategies are essential to reduce the consequent burdens. In terms of green chemistry and ecological sustainability, enzymatic bioremediation is by far the safest and most efficient route for the transformation of xenobiotics to simpler, non-malicious molecules (Bharadwaj, 2018).

Laccases (EC 1.10.3.2), have attracted the attention of researchers due to their ability to catalyze mono-electronic oxidation of phenolic substrates, requiring only atmospheric molecular oxygen as their co-substrate (Kumar et al., 2014). However, the catalytic activity of laccase is assumed to occur *via* the loss of an electron by the reducing substrate, resulting in the formation of an oxygen-centered cationic radical, followed by either subsequent rounds of oxidation or polymerization, ultimately resulting in quinone formation and/or partial precipitation (Janusz et al., 2020). In xenobiotics, such as pesticides, the presence of a phenolic ring structure makes laccase a viable option for its remediation (Bilal et al., 2019). Due to its broad range of substrate specificity and ability to catalyze the breakdown of non-phenolic molecules in the presence of mediator systems, this enzyme is an interesting candidate to study for its potential for the bioremediation of pesticide-contaminated wastewaters (Sathishkumar et al., 2019).

In our previous studies on the laccase activities of various fungi, *Pleurotus dryinus* showed remarkably high laccase activity in submerged fermentation (SmF) and was found to be capable of removing phenolic compounds and polycyclic aromatic hydrocarbons (Batista-García et al., 2017; Ariste et al., 2020). Although the laccases from *P. dryinus* have already been produced in SmF (Ariste et al., 2020), there is still the potential to increase laccase production. Solid-state fermentation (SSF), a cost-efficient process, uses solid substrates derived from agricultural by-products and residual wastes, which also act as a natural habitat by supporting the growth of the microorganisms and laccase production (Wang et al., 2019; Xu et al., 2020). Rodríguez-Couto (2018) reviewed the inert lignocellulosic biomass-based support substrates such as barley bran, sugarcane bagasse, corncob, oat straw, rice straw, and wheat straw used by white-rot fungi (WRF) for the production of laccase enzymes under SSF conditions (Rodríguez-Couto, 2018). Osma et al. (2011a) found that the final cost of the laccase production under SSF conditions was 50 times lower than the final cost of the laccase production under SmF conditions (Osma et al., 2011b). Thus, the main purpose of this research is to investigate the possibility of developing cost-effective SSF using *P. dryinus* as the substrate to produce laccase.

To reduce these costs, sludge/biosolids (BS) are used as support substrates in SSF for organic waste valorization (Abu Yazid et al., 2017). Recently, anaerobic digestate from the biogas unit was valorized *via* SSF for cellulase and xylanase production using *Trichoderma reesei,* a biosurfactant from *Starmella bombicola* and biopesticides from *Bacillus thuringiensis* (Mejias et al., 2018; Cerda et al., 2019). BS are heterogenous solid matrices derived from wastewater treatment plants (WWTPs) consisting of bacterial constituents such as cellulose, hemicellulose, proteins, lipids coupled with other organic matter (OM) and inorganic matter, pathogens, and contaminants (Barnabé et al., 2009). Because they are readily available, BS are currently used by the agricultural industry for soil amendment (Sharma et al., 2017). However, the increasing number of WWTPs poses serious problems in terms of BS management and disposal. Even though BS have a high N and P content, the presence of heavy metals in the BS can adversely affect plants and lead to food chain contamination if excessive amounts of it are spread over agricultural soils (Sharma et al., 2017). Since most of the agricultural runoff ultimately joins freshwater sources, an initial study investigating the effectiveness of the laccase to catalyze the transformation of the potent pesticide contaminants was necessary to consider its applicability and further development into a viable bioprocess.

Hence, the two main objectives of the study are 1) to produce laccase by fermentation (BS-based crude enzyme extract) by bioaugmenting *P. dryinus* in pretreated and untreated BS and 2) to evaluate the efficiency of BS-based crude enzyme extract by comparing the batch treatment of a cocktail of 29 pesticides with BS-based crude enzyme extract and commercial laccase. These pesticides were chosen for the study based on their global prevalence and their documented adverse effects on ecological systems and human health (Yadav and Devi, 2017). To the best of our knowledge, such a diverse work of this magnitude investigating the elimination of herbicides, fungicides, and insecticides in an aqueous solution using laccase has not been previously published.

## 2 Materials and Methods

### 2.1 Chemicals

All the pesticides (≥98% purity) and extraction reagents were of analytical grade and purchased from Sigma-Aldrich (Saint Louis, MO, United States). Laccase from *Trametes versicolor* (>10 U/mg) was obtained from Fluka (Buchs, Switzerland). 2, 2′-azino-bis (3-ethylbenzothiazoline-6-sulphonic acid) was purchased from Sigma-Aldrich. The UHPLC-grade methanol used to prepare pesticide stocks was purchased from Thermo Fisher Scientific (Waltham, MA, United States). The deionized Milli-Q ultrapure water from the EMD Millipore water filtration system (Billerica, MA, United States) was used in the study.

### 2.2 Pesticide and Enzyme Stock Preparation

About 10 ppm pesticide stocks (See Table 1) were prepared by dissolving the required amount of the respective compounds in an HPLC-grade methanol solution in glass vials. The vials were covered with aluminum wrapping to prevent any inadvertent photochemical degradation of the pesticides in the methanol solution. Free laccase was added to 0.1 M pH 4.0 acetate buffer to a stock concentration of 10 mg/ml and stored at 4°C before use.

**TABLE 1:** List of pesticide (herbicide, fungicide, and insecticide) compounds tested in this study.

Xenobiotics		Molecular weight (g/mol)	Log k_ow_
Fungicide	Carbendazim	191.19	1.52
	Thiabendazole	201.25	2.47
	Pyrimethanil	199.26	2.84
	Kresoxim methyl	313.3	3.4
	Pyraclostrobin	387.8	3.99
	Trifloxystrobin	408.37	4.5
	Boscalid	343.2	2.96
	Iprodione	330.16	3
	Fludioxonil	248.18	4.12
Herbicide	Diuron	233.09	2.68
	Simazine	201.65	2.18
	Monolinuron	214.6	2.3
	Atrazine	215.68	2.61
	Hexazinone	253.32	1.85
	Pendimethalin	281.31	5.2
	Metolachlor	283.79	3.13
	Imazethapyr	289.33	1.49
	Metobromuron	259.1	2.4
	Bentazon	240.28	2.34
Insecticide	Aldicarb	190.27	1.13
	Carbofuran	221.25	2.32
	Acetamiprid	222.67	0.8
	Parathion	291.3	3.83
	Azinphos-methyl	317.32	2.75
	Chlorpyrifos	350.59	5
	Malathion	330.4	2.36
	Coumaphos	362.77	4.13
	Chlorfenvinphos	359.6	3.81
	Spinosad	732	2.8

### 2.3 Laccase Production by Fermentation in Shake Flasks

The BS and wastewater effluent (WWE) samples were collected from a local municipal WWTP (Quebec, Canada) and transported to the laboratory for processing. The BS samples were stored in a freezer at −18°C, and the WWE samples were stored in a refrigerator at 4°C before use. Before beginning the initial characterization and fermentation process, the frozen BS were thawed at 37°C for 12 h and then the free water was decanted. The resulting BS were used for the main initial characterization (Table 2). Given the variability of BS, the standard deviation in this table corresponds to the average of more than four samples analyzed for 1 year and collected from the municipal WWTP.

**TABLE 2:** Characterization of biosolids (BS).

Parameters	Value
pH	6.8 ± 0.3
Total Carbon (mg/g)	310.7 ± 9.1
Total Hydrogen (mg/g)	38.9 ± 1.8
Total Nitrogen (mg/g)	48.6 ± 0.7
Total Phosphorus (mg/g)	3.1 ± 0.2
C: N ratio	6.39
COD (mg/ml)	13.1 ± 1.2
TSS (mg/ml)	74.9 ± 5.3
TDS (mg/ml)	4.9 ± 0.2
VSS (mg/ml)	48.6 ± 2.3
Total proteins (mg/g)	9.8 ± 0.4
Total reducing sugar (mg/g)	0.9 ± 0.1

#### 2.3.1 Inoculum Preparation

*P. dryinus* IBB903, a basidiomycetes, was obtained from the Institute of Biochemistry and Biotechnology, Georgia. Upon collection, the strain was grown in Petri dishes with GYM agar [glucose (0.5% (w/v)], yeast extract [0.75% (w/v)], and malt extract [0.25% (w/v)] under sterile conditions. Then, three plugs (diameter 3 mm) of *P. dryinus* cultured in GYM agar plates was taken and inoculated in a 250 ml culture flask containing 100 ml sterilized GYM media for 5 days (20°C and at 150 rpm). Then, the fungal broth solution was filtered and the biomass was rinsed and then blended with sterile saline solution and stored at 4°C (Haroune et al., 2014). Then, 1 ml mycelium solution was transferred into 100 ml of sterilized WWE supplemented with GYM media in a 250 ml flask. This was cultured for 5 days and used as the inoculum throughout the study. The pH was adjusted to 6.0 using 0.1 M HCl, and all cultures were incubated at 20°C.

#### 2.3.2 Preparation of BS for Fermentation Experiments

One of the main objectives of this study was to evaluate the potential of BS for laccase production. So, in this study, unsterilized biosolids (UBS) were screened along with three other forms of biosolids: hygenized biosolids (HBS), sterilized biosolids (SBS), and alkali-treated biosolids (ABS) for the production of laccase under SSF for up to 21 days. For UBS to produce laccase in SSF, unsterilized BS and WWE were used. To use SBS as a substrate for laccase production in SSF, the BS and WWE were initially sterilized in an autoclave at 121°C and 15 psig (103.421 kPa) for 15 min. For HBS, BS and WWE were kept for 1 h in a preheated oven at 70°C and used as a substrate for laccase production in SSF. For ABS, BS was mixed with unsterilized WWE and incubated in an alkaline condition (pH 12.4) using 50% sodium hydroxide for 4 h. After incubation, the digested samples were centrifuged at 1,520 x g for 30 min to obtain the pellet and supernatant. The pellet was used for further SSF, while the supernatant was discarded. The BS produced from different processes were stored in a cold room at 4°C for not more than 2 weeks before fermentation.

#### 2.3.3 Characterization of BS for Laccase Production

Fermentation experiments were performed using the treated BS (SBS, UBS, HBS, ABS) as a substrate and a sponge cloth (Scotch Brite; 3M, Maplewood, MI, USA) as the bulking agent. The BS, used as the substrate in this study, had a high moisture content and low porosity (Shammas and Wang, 2009), two factors that limit the oxygen transfer in the solid matrix. In order to promote oxygen transfer and provide adequate porosity, inert material (sponge cloth) was cut into small pieces and added as a bulking agent during fermentation (Cerda et al., 2019). The ratio of the substrate to the bulking agent was 90:10 (w/w). Fermentations were carried out in triplicates in 0.25 L Erlenmeyer flasks with a total weight of 50 g of material in each flask. This was prepared by adding 45 g of treated BS and 5 g of sponge cloth, followed by mixing 10 ml of sterile WWE to maintain the humidity. Moistened BS were then transferred to flasks, and the flasks were plugged with cotton and autoclaved at 121°C for 15 min. To ensure that there was no contamination, each flask was inoculated with 2 ml of actively growing *P. dryinus* cultured in sterilized WWE supplemented with GYM media for laccase production experiments. To prevent evaporation, the flasks were kept in complete darkness in a static incubator at 30°C, and 90% humidity. In addition to routine analysis, including dry matter, moisture content, and pH, sampling was taken every 24 h of fermentation by removing 2 g of the content of each flask to continuously monitor total protein (Rathankumar et al., 2020), total reducing sugar (Rathankumar et al., 2020), chemical oxygen demand (Vaithyanathan et al., 2021a), total suspended solids (Vaithyanathan et al., 2021b), volatile suspended solids, total dissolved solids (Vaithyanathan et al., 2021b), ligninolytic enzymes like laccase (Touahar et al., 2014), lignin peroxidase (Lip; Ariste et al., 2020), aryl alcohol oxidase (AAO; Ariste et al., 2020), and cellulase (Vaithyanathan et al., 2021a). Enzyme activities were measured spectrophotometrically using a double beam SpectraMax Plus 384 UV-Vis spectrophotometer (Molecular Devices Corporation, Sunnyvale, CA, USA). One unit of enzyme activity was defined as the amount of enzyme required to oxidize 1 µmol of substrate per minute. Assays were carried out in triplicates, and the average of the three was reported.

#### 2.3.4 Extraction of Enzymes

To maximize the amount of laccase recovered, ultrasonication studies were performed in an ultrasonication water bath (VWR Symphony-TM, Edmonton, AB, Canada) to recover the enzyme associated with the cell and membrane of *P. dryinus* and the flocs formed in the BS. The Day 12SBS production medium inoculated with *P. dryinus* was sonicated at 5-min intervals over a 30-min period. After sonication, the production medium was centrifuged at 1,520 x g for 10 min at 4°C; then, the supernatant was separated and then filtered (0.7 μm glass fiber filter) and tested for enzyme activity and total protein.

### 2.4 Pesticide Removal From a Secondary Effluent

In January 2020, a sample of biologically treated wastewater was collected from the effluent of the local municipal WWTP in Quebec, Canada. The pH (originally 7.4) was adjusted to pH 7.0 with acetic acid 1 M and then spiked with each of the tested pesticides to attain initial concentrations in the range of 10 μg/L. The 29 pesticides were tested by commercial *T. versicolor* laccase and BS-based *P. dryinus* crude enzyme extract in two different kinds of experimental sets. In the first set, each of the 29 pesticides was individually tested by a crude enzyme extract and a commercial laccase. In the second set, a cocktail of 29 pesticides was tested by a crude enzyme extract and a commercial laccase.

All experiments were performed in batch mode at 30°C in 50 ml Erlenmeyer flasks with orbital shaking at 150 rpm for 24 h. The final volume of samples was 10 ml. Crude laccase extract and commercial laccase were added to obtain a final laccase activity of 1,000 U/L. For the controls, the biocatalyst solutions were substituted with deactivated crude laccase extract or phosphate buffer solution. Crude enzyme extract was heated up to 120°C for 20 min to collect the deactivated enzyme (Xu et al., 2020). After reaction times varying from every 6 to 24 h, the reaction mixture was filtered by using 0.2 μm PTFE membrane, and the pesticides were extracted before analysis by ultra-performance liquid chromatography coupled with tandem mass spectrometry (UPLC-MS/MS). For each compound, the enzymatic removal by crude laccase extract was calculated according to the concentration detected in the controls with inactivated laccase and without enzyme, respectively. The control without enzyme was used as a reference to calculate the total removal of each compound by laccase. All experiments were carried out in triplicates, and the results reported are the arithmetic averages of the three with their standard deviation statistical analysis; one-way ANOVA was performed to check the significant difference using Microsoft Excel.

### 2.5 Extraction and Quantification of Pesticides

A previously developed liquid–liquid extraction technique and sample preparation protocol for ultra-performance liquid chromatography (UPLC) analysis was slightly modified and employed as follows (Touahar et al., 2014). Samples from the batch reaction were transferred to a separation funnel and added to the excess NaCl and shaken well to dissolve the salt. About 5 ml of ethyl acetate was added to the solution and mixed well. Then, the resulting solution from the organic phase was collected in a glass vial without any water droplets. To maximize the recovery of the pesticide residue, a second round of extraction was performed by adding 5 ml of methylene chloride to the previously obtained aqueous phase and mixing it well. Once again, the resulting solution from organic phase was collected in the same vial, and then the liquid was dried by evaporation under a stream of flowing nitrogen gas. The residue in the dried vial was resuspended in a 50%–50% (v/v) solution of water and methanol of HPLC grade and 0.1% (v/v) formic acid. The solution was vortexed and then ultrasonicated to ensure a complete dissolution of the pesticide residue. The solution was filtered by passing it through a 0.2 µm PTFE membrane before transferring it to amber glass vials for analysis. The samples were stored at 4°C until further quantitative analysis by the Waters Acquity UPLC-MS/MS system (Milford, MA, United States) using a previously published protocol (Touahar et al., 2014; Haroune et al., 2015; Vaithyanathan et al., 2021a).

## 3 Results and Discussions

The selection of a cost-effective substrate is a major factor for the production of laccase by WRF fermentation through SSF (Osma et al., 2011b). BS are mainly comprised of OM (45%–70%), 3%–8% nitrogen and other essential nutrients like calcium, sulfur, phosphorus, magnesium, and potassium, These nutrients play an important role in the growth of plants and microorganisms (Sullivan et al., 2015). The results of the BS mixture characterization (Table 2) showed that BS contain significant amounts of nutrients required for the survival of microorganisms; hence, it was hypothesized that they could be used for lignolytic enzyme production. BS are a preferred choice for laccase production since they promote the growth and establishment of the fungus within raw material and act as an additional carbon source for the WRF that can be easily metabolized to degrade organic compounds in raw materials. To avoid any nutritional variation, all the experiments were performed using the same BS batch sample.

### 3.1 BS Fermentation for Laccase Production

Due to its complex molecular structure, the OM in the BS is not readily bioavailable. In order to break down those hardly degradable components, pretreatment (PT) was applied to BS, which converts those complex OM into easily assimilable components and make these substrates easily usable for microorganisms during the biological process (Vaithyanathan et al., 2021a; Vaithyanathan and Cabana., 2021). The purpose of the PT in BS was not only to break down complex molecules, but also to be involved in other applications such as pathogen reduction, improving biogas yield, the extraction of value-added products, reduced retention time in digesters, solid content reduction, etc. (Vaithyanathan and Cabana., 2021).

**FIGURE 1:**
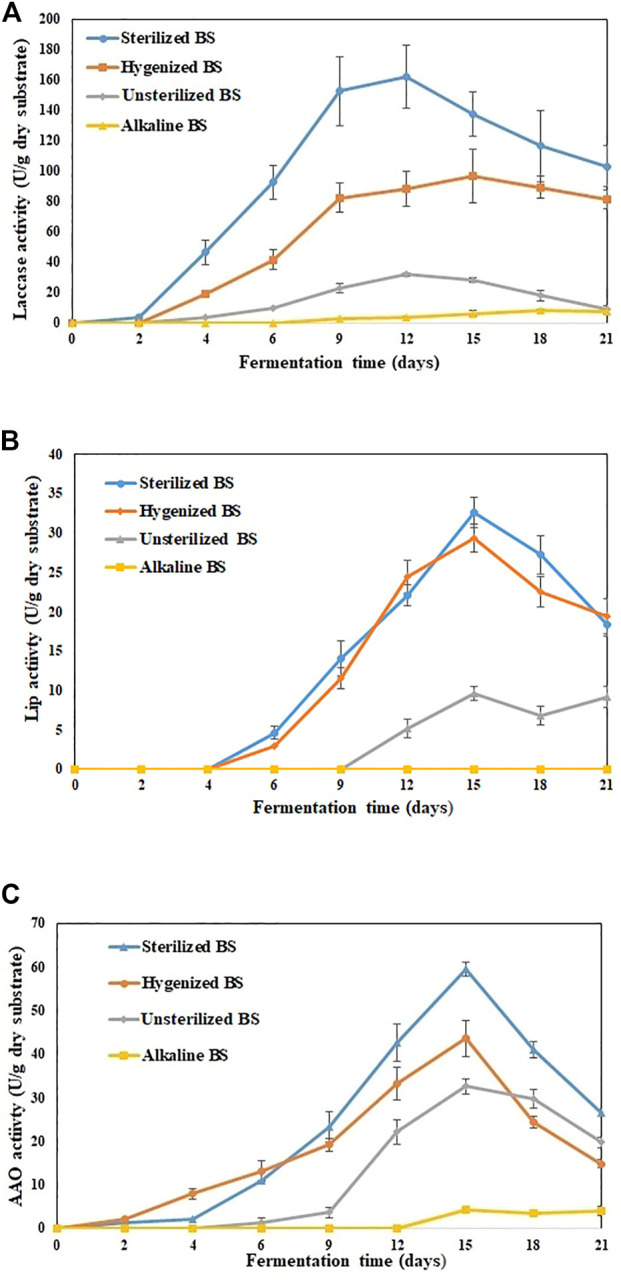
Extracellular enzyme activities detected in various treated BS during 21 days of fermentation **(A)** laccase **(B)** Lignin peroxidase **(C)** Aryl alcohol oxidase. The experiments were done in triplicate at pH 7.0 and 30°C, and the error bar represents the standard deviations in each set of determinations.

In this research, various types of BS PT for producing lignolytic enzymes under SSF were studied up to 21 days. During the early stage of the experiment (first 7 days), PT improved the availability of adequate nutrient content, so the fungi inoculated in SBS and HBS used these nutrients in the culture medium for spore germination and hyphal growth (Li et al., 2019). While the fermentation continued, by the end of Day 15, the medium was entirely colonized by fungi. However, for UBS, the visible growth of fungi was only observed after Day 12, and by the end of Day 21, fungi colonization on the surface was just under a quarter. Figure 1 shows the trend of laccase activity with the fermentation time, suggesting that *P. dryinus* was able to use BS as a carbon source and support for mycelium growth, as well as for the metabolic secretion of laccase.

SBS was the most promising substrate for laccase production by *P. dryinus* with the maximum laccase activity of (162.1 ± 21.1 U/g dry substrate; Day 12 of fermentation), followed by HBS (96.7 ± 17.6 U/g dry substrate; Day 12 of fermentation), UBS (31.9 ± 1.2 U/g dry substrate; Day 12 of fermentation), and ABS (8.2 ± 0.4 U/g dry substrate; Day 18 of fermentation). Interestingly, the laccase activity results indicated a significant difference (*p* < 0.05) between not-pretreated biosolids (UBS) and pretreated biosolids (SBS, HBS, and ABS), which also implies that PT has an impact on the substrate, which in turn increases or decreases the laccase activity in BS when compared with no PT. The longest laccase production period and highest laccase yield were observed in SSF while using SBS and HBS. During Day 12, the maximum laccase activity (162.1 ± 21.1 U/g dry substrate) obtained with SBS was 5.1 times higher than that obtained with UBS and 1.8 times higher than that obtained with HBS (Figure 1A). In SBS, the rate of change in enzyme activity linearly increased from Day 1 (1.6 U/g/day dry substrate) to Day 6 (23.1 U/g/day dry substrate); then, it reduced slightly by Day 9 (20 U/g/day dry substrate). After Day 9, the rate of change in enzyme activity decreased drastically until the end of the experiment.

For HBS and UBS, the rate of change in enzyme activity increased linearly until Day 9 vs. Day 6 for SBS. Then, after Day 9, the rate of change in enzyme activity decreased slowly for UBS, whereas for HBS, it remained similar to that obtained with SBS. The improved enzyme activity observed during sterilization and hygenization of BS implies that the hydrolysis of OM or the degradation of components occurred faster when BS was subjected to heat treatment (Sousa et al., 2018). ABS induced laccase activity after Day 9; by the end of the experiment, the activity was 7.1 ± 1.1 U/g dry substrate, which was 23, 92, and 94% less than for UBS, HBS, and SBS, respectively, during the same period. Subjecting BS to a high pH for a long duration disrupts the floc structure and release of high molecular components, surface-active agents, and other intermediate products. As a result, it creates an unfavorable condition for the fungi to acclimatize during their growth phase, which subsequently affects enzyme production (Vaithyanathan et al., 2021a). The type of substrate will affect the growth of *P. dryinus* in the SSF process, which also directly affects the quality of the laccase produced. The laccase production of 162 U/g is in the higher range reported at laboratory-scale SSF using sterile substrates or adding a single strain (Osma et al., 2011a; Jimenez et al., 2019).

The enzymes that are produced could remain immobilized in the solid substrate and hence result in a low enzymatic content in the BS mixture. The substrate structure is altered during the SSF performance, i.e., more accessible, and simple hydrolysates are generated. These hydrolysates can be of any origin such as proteins, lipids, or carbohydrates and therefore do not imply improved laccase production. Apart from laccases, other enzymes such as Lip and AAO were also found in treated BS. On Day 15, the maximum Lip (32.6 ± 1.9 U/g dry substrate), AAO (59.4 ± 1.6 U/g dry substrate), and cellulase (52.1 ± 3.4 U/g dry substrate) activities were observed in SBS, which were 3.3-fold, 1.8-fold, and 1.4-fold higher than UBS for the same period (Figures 1B–D). For LiP and AAO, a significant difference (*p* < 0.05) was observed between non-pretreated biosolids (UBS) and pretreated biosolids (SBS and ABS). On the other hand, no significant difference (*p* > 0.05) was observed between UBS and HBS.

### 3.2 Enzyme Recovery Using Ultrasonication

**FIGURE 2:**
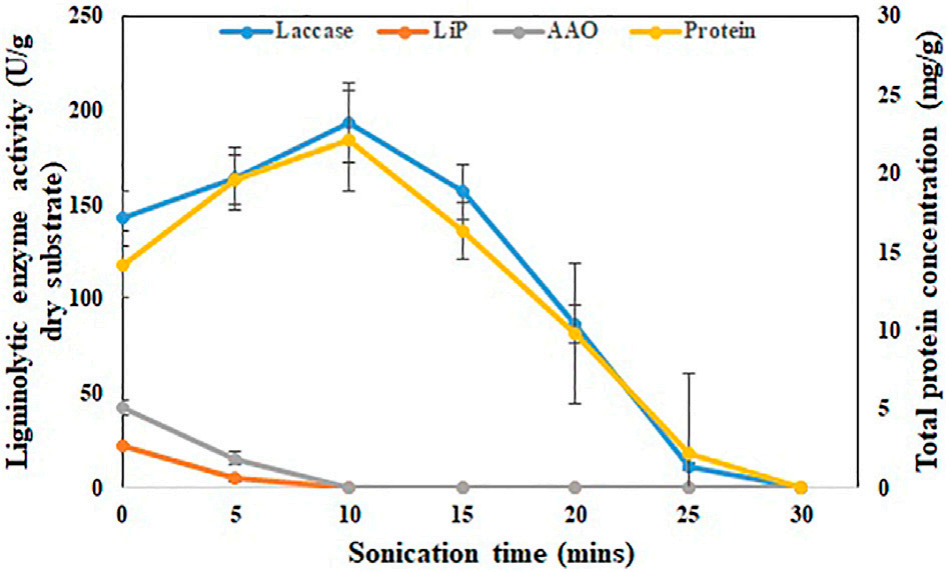
Effect of ultrasonication on recovery of laccase from SBS. Enzyme activity (U/g) and protein concentration (g/L) with respect to time during sonication for 30 min. The experiments were done in triplicate and the error bar represents the standard deviations in each set of determinations.

SBS production medium, which obtained the maximum laccase yield (162.1 ± 21.1 U/g dry substrate) compared to other treatments such as ABS, UBS, and HBS, will be used for further enzyme recovery and pesticide degrading experiments. Sonicating the SBS production medium in an ultrasonic bath for 10 min increased the enzyme activity to 193.2 ± 13.2 U/g dry substrate from 142.6 ± 14.1 U/g dry substrate (Figure 2). During the same period, the protein concentration also increased from 14.2 ± 0.5 g/L to 19.6 ± 1.3 g/L (Figure 2). During 25 min of sonication, the enzyme activity further decreased to 11.4 ± 0.2 U/g dry substrate after 10 min. The enzyme recovery rate during the first 5 min (163.7 U/g dry substrate) was 4.22 U/g/min and then increased to 5.9 U/g/min for the period from 5–10 min (193.2 U/g dry substrate). After 10 min, the enzyme recovery rate decreased drastically in the rate of 7.26 U/g/min (156.9 U/g of dry substrate), 14.02 U/g/min (86.8 U/g of dry substrate), and 15.08 U/g/min (11.4 U/g of dry substrate) for 15, 20, and 25 min sonication, respectively. The maximum laccase activity of 193.2 ± 13.2 U/g dry substrate achieved during 10 min of sonication was 26.1% higher than the initial activity (Figure 2). On the other hand, other ligninolytic enzyme activities like lignin peroxidase and AAO were severely impacted by sonication. Due to sonication exposure, lignin peroxidase and AAO were not detected after 5 min. The maximum lignin peroxidase (4.9 ± 1.7 U/g of dry substrate) and AAO (15.6 ± 3.7 U/g of dry substrate) after sonication were 78 and 63% lower than the initial activity (Figure 2).

In basidiomycetes, like *P. dryinus*, the extracellular cellular enzyme produced during fermentation closely binds to the mycelial hyphal structure as their interlinkage association is strong (Bentil et al., 2018). In addition, fungi that grow over BS form an agglomeration, which generates a floc-like formation where the secreted enzymes immobilize to it (Vaithyanathan et al., 2020). The enzyme binds strongly to the substrate during hydrolysis, making it difficult to recover by normal washing (Bentil et al., 2018). Sonication, which generates hydroxy radicals, plays a major role in breaking the hyphal-floc structure and improving the enzyme activity. However, prolonged exposure of BS to sonication time increases the temperature results in the deactivation of the enzyme, which was the probable cause of the decrease in the enzyme activity (Vaithyanathan et al., 2020).

### 3.3 Comparative Assessment of the Remediation of Pesticide-Spiked Wastewater Effluent by the Recovered Crude Laccase Extract and Commercial Laccase

**FIGURE 3:**
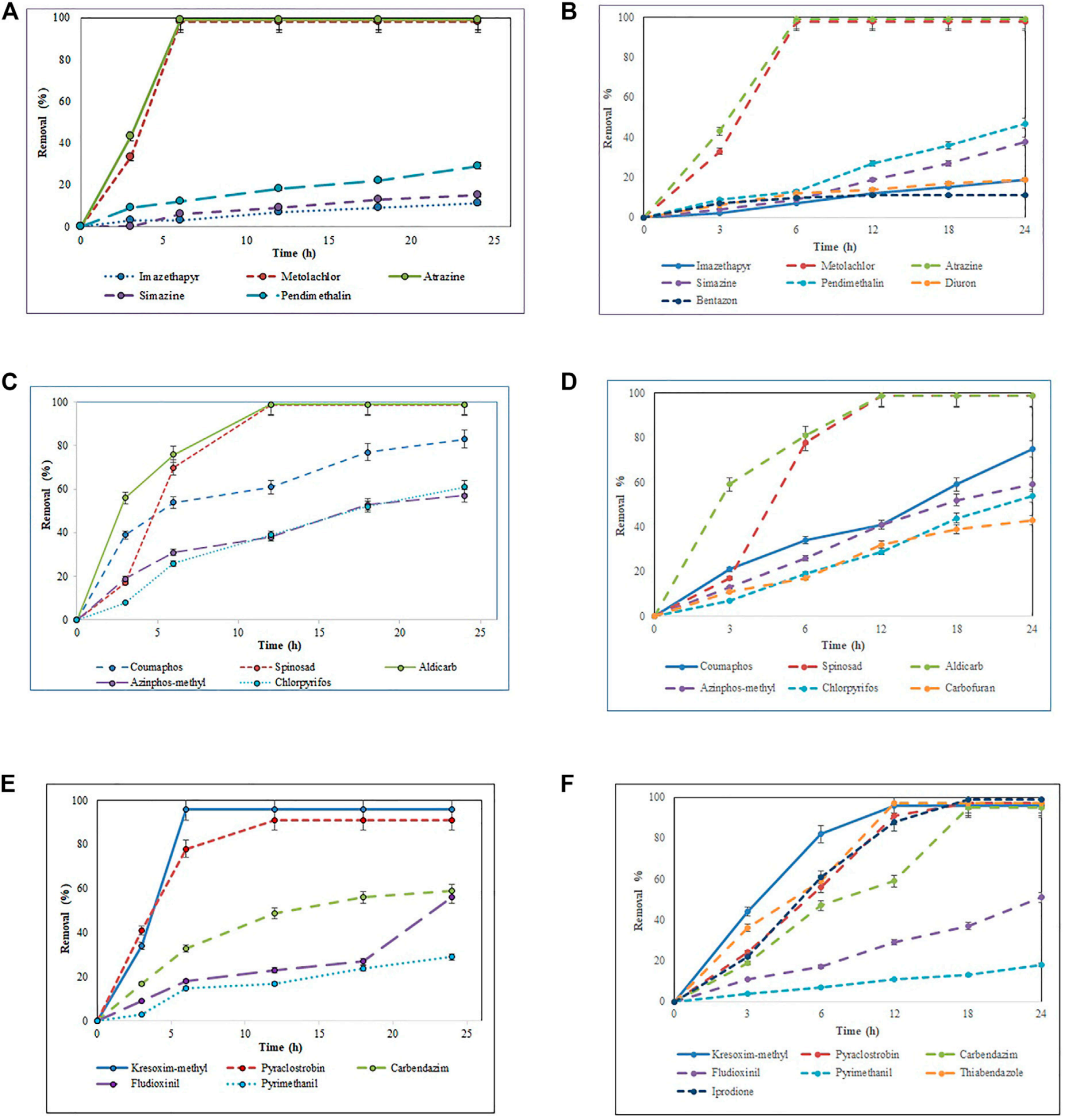
Bio-catalytic removal of **(A)** 10 herbicides, **(C)** 10 insecticides, **(E)** nine fungicides treated separately in aqueous solution by *Pleurotus dryinus* laccase derived from BS and removal of **(B)** 10 herbicides, **(D)** 10 insecticides, **(F)** nine fungicides treated separately in aqueous solution by commercial laccase as a function of time. The experiments were done in triplicate at pH 7.0 and 20°C, and the error bar represents the percentage error at 5% level of significance.

**FIGURE 4:**
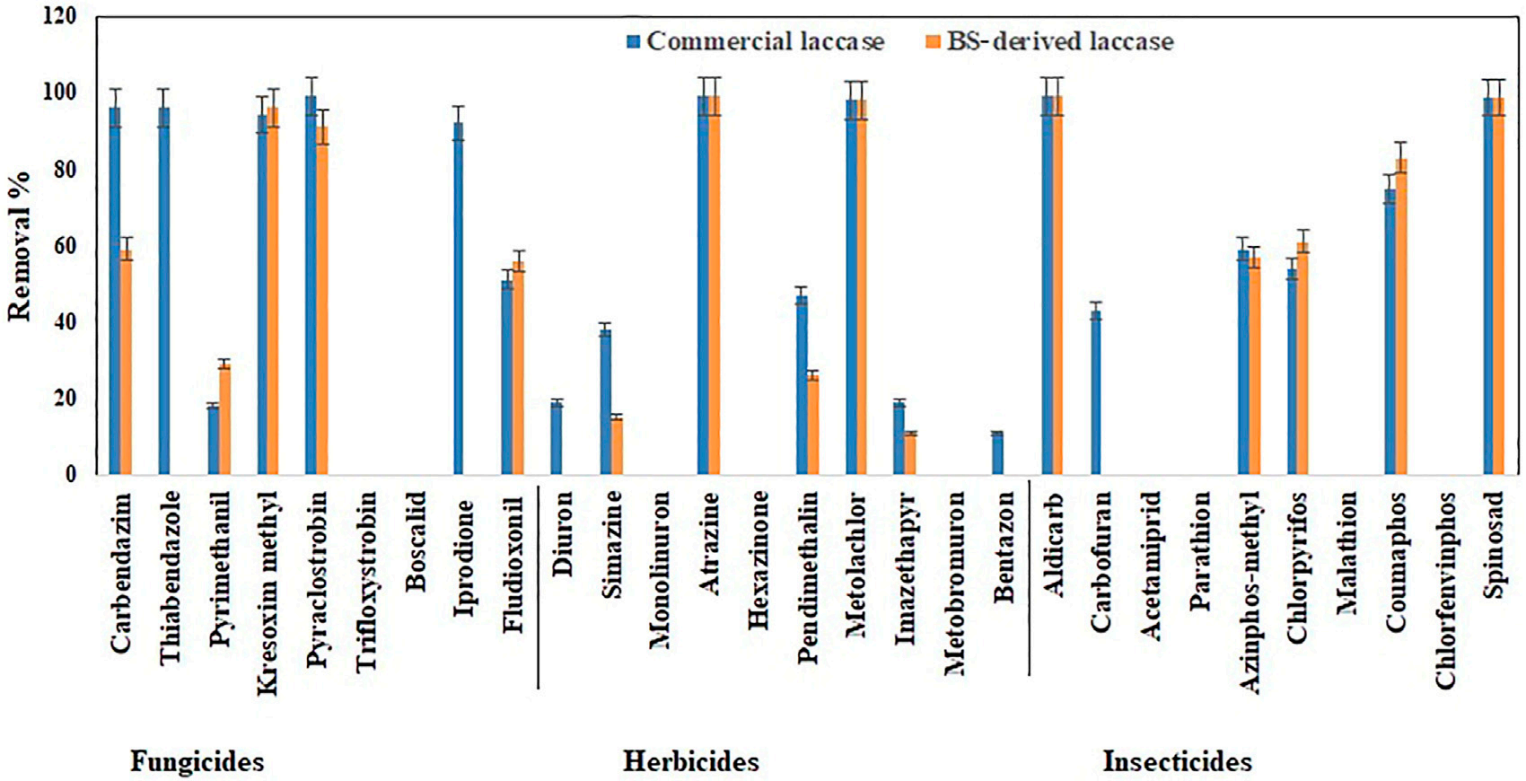
Bio-catalytic removal of 29 pesticide compounds treated separately in aqueous solution by *Pleurotus dryinus* laccase derived from BS and commercial laccase during 24 h. The experiments were done in triplicate at pH 7.0 and 20°C, and the error bar represents the percentage error at 5% level of significance.

**FIGURE 5:**
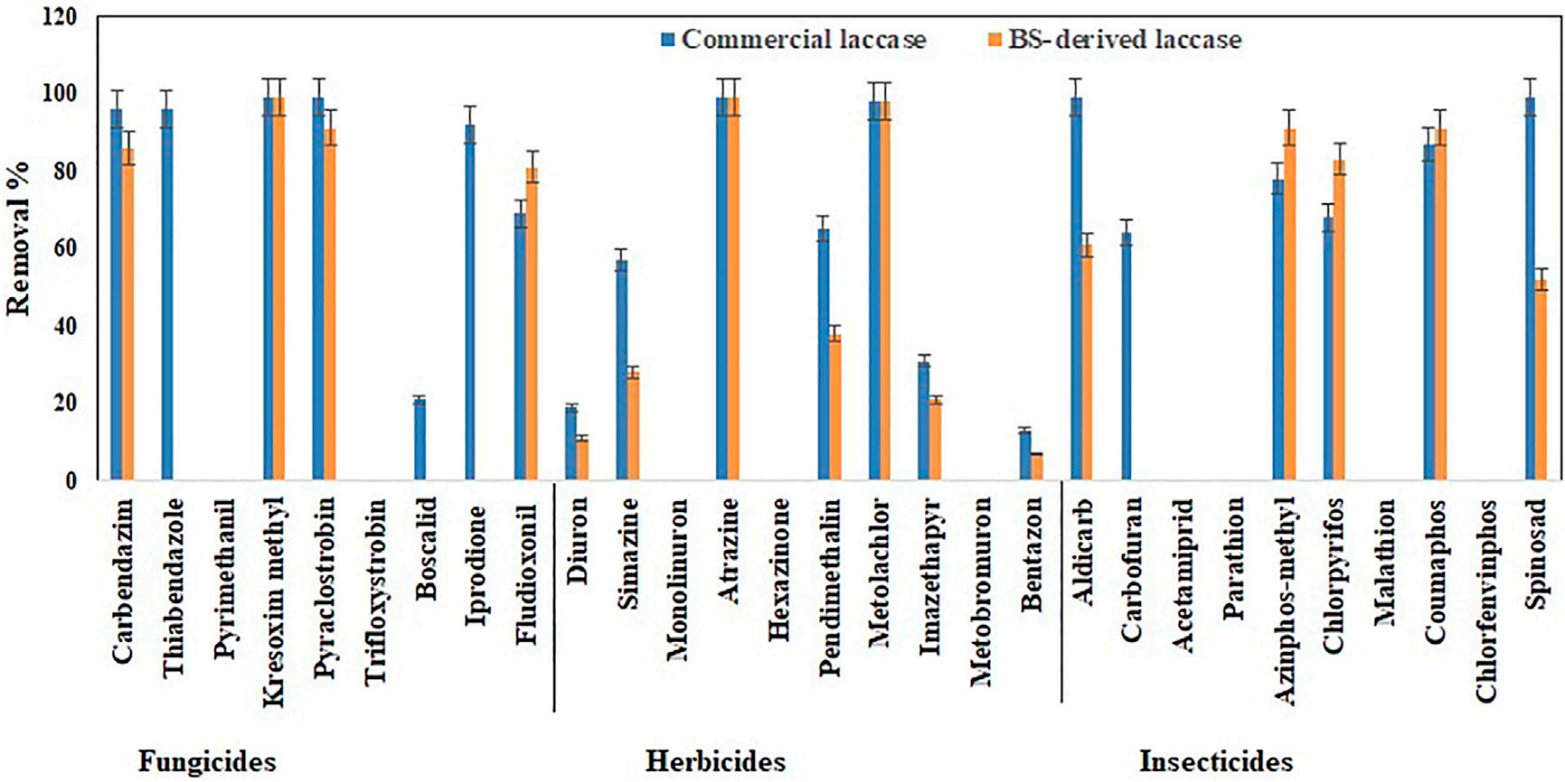
Bio-catalytic removal of 29 pesticide compounds treated as a cocktail mixture in aqueous solution by *Pleurotus dryinus* laccase derived from BS and commercial laccase during 24 h. The experiments were done in triplicate at pH 7.0 and 20°C, and the error bar represents the percentage error at 5% level of significance.

The total pesticide removal percentages for BS-derived crude enzyme extract and commercial laccase in cocktail after 24 h were 35.8 and 49.9%, while the total pesticide removal percentages after 24 h when treated individually were 33.7 and 44.8% for BS-derived crude enzyme extract and commercial laccase respectively. Of the 29 pesticides tested in each set of experiments, eight pesticides were completely removed with commercial laccase vs. five pesticides with BS-derived laccase. On the other hand, 9 pesticides remained recalcitrant with commercial laccase and 13 with BS-derived laccase. Figures 3–5 show the elimination of herbicides, insecticides, and fungicides in the comparative study with individual batches and as a cocktail.

#### 3.3.1 Removal of Herbicides

In separate flask experiments, herbicides like atrazine and metolachlor were almost completely removed within the first 6 h when treated with BS-derived crude enzyme extract and commercial laccase (Figures 3A,B). Similarly, Jin et al. (2016) reported that over 8 days of degradation studies, 91.6% of atrazine was removed from *T. versicolor* when treated with commercial laccase. During 24 h of the experiment, pendimethalin (47%), imazethapyr (19%), and simazine (38%) were partially removed when treated with commercial laccase vs. 26, 11, and 15%, respectively, with BS-derived crude enzyme extract (Figure 4). Out of 10 herbicides tested, 5 herbicides, namely, monolinuron, hexazinone, diuron, metabromuron, and bentazon, remained recalcitrant with BS-derived crude enzyme extract, and only 3 herbicides (monolinuron, hexazinone, and metabromuron) remained recalcitrant with commercial laccase. However, when diuron and bentazon were treated with commercial laccase, they were partially removed (19% for diuron and 11% for bentazon) after 24 h (Figure 4) and a major part of this removal (12% for diuron and 10% for bentazon) occurred during the first 6 h of treatment (Figure 3B). When comparing the herbicide removal efficiency of commercial laccase to that of the BS-derived crude enzyme extract, a significant difference (*p* < 0.05) was only observed for diuron, bentazon, and imazethapyr.

Apart from laccase, other ligninolytic enzymes were not observed in the recovered BS-derived crude enzyme extract, so pesticide degradation during this study may be the result of laccase enzymes. Although it is still not known exactly how laccase catalyzes the initial monoelectronic oxidation of its primary substrate to the complex transformation of phenolic xenobiotics to relatively simpler and non-toxic molecules, several hypotheses exist for the catalytic disruption of bonds by laccase in its target molecule. These involve Cα oxidation, Cα-Cβ cleavage, which causes the separation of linear organic chains from the aromatic ring, and aryl alkyl cleavage, which causes the removal of substituent functional groups from the aromatic ring structure, all of which result in the formation of cationic phenols that either polymerize to precipitate out of the solution, form a quinone, or undergo subsequent rounds of oxidation to form simpler compounds (Yaropolov et al., 1994; Kunamneni et al., 2007). In general, the ability of laccase to catalyze oxidation is improved if the redox potential of the substrate is low (Cañas and Camarero, 2010). The presence of functional groups as substitutions in vital positions of the aromatic ring makes the substrate more susceptible to oxidative attack by laccase (Nguyen et al., 2014; Touahar et al., 2014; Kumar and Cabana, 2016).

Interestingly, when these herbicides were treated as a cocktail, the removal percentage of pendimethalin (37%), imazethapyr (21%), simazine (28%), bentazon (7%), and diuron (11%) increased around 7%–13% with BS-derived crude enzyme extract (Figure 5). However, with the exception of bentazon and diuron, no significant difference (*p* > 0.05) was observed in the removal percentages of herbicides in the cocktail and isolated experiments with the BS-derived crude enzyme extract. This phenomenon of increased catalytic transformation of certain substances by laccase in the presence of other similar molecules could possibly be due to the laccase mediator effect, in which an oxidized substrate molecule acts as a secondary substrate to drive the monoelectronic oxidation of the primary substrate (Bourbonnais and Paice, 1990). With commercial laccase, the removal percentage improved by 18% for pendimethalin, 11% for imazethapyr, and 19% for simazine (Figure 5). On the other hand, the removal of diuron and bentazon with commercial laccase remained unchanged or changed a little\. Diuron (19%) and bentazon (11%) removal obtained by commercial laccase tested in a herbicide mixture during this study were very low when compared with the diuron (63%) and bentazon (74%) removal percentages obtained by commercial laccase from *T. versicolor* in the Kupski et al. (2019) study.

Atrazine and metolachlor were completely removed with both sources of laccase. Similar results were also obtained with separate flask experiments. Laccase drives the transformation of pesticides *via* the dealkylation of aromatic rings, the cleavage of the thioether bond, and the oxidative removal of any sulfur-containing moieties. The phosphorothiolate group of organophosphorus pesticides, such as those considered in this study, along with chloroperoxidases from fungi are initially oxidized to produce oxon derivatives, in which the P-S bond in the parent molecule is replaced by a P-O bond to form a phosphate (Amitai et al., 1998; Torres et al., 2003). Although oxon derivatives are usually more toxic than organophosphorus compounds, the presence of other related compounds that act as laccase mediators facilitates the detoxification of these compounds.

#### 3.3.2 Removal of Insecticides

Except spinosad and aldicarb, the eight other insecticides tested in this study were either partially removed or remained recalcitrant. Figures 3C,D, 4, 5 show the results when insecticides where subjected to reacting with BS-derived crude enzyme extract and commercial laccase separately and as a cocktail for 24 h. In a separate flask experiment, the maximum removal of BS-derived crude enzyme extract was obtained for aldicarb (99%), and Spinosad (98%), followed by coumaphos (83%). Chlorpyrifos (61%) and azinphos-methyl (57%) were partially removed (Figure 4). Chlorpyrifos removal with *P. dryinus* laccase grown over BS as a substrate was higher than with laccase purified from *Tricholoma giganteum* AGDR1 with wheat straw as a substrate (29%) (Rudakiya et al., 2020). The maximum removal with commercial laccase was aldicarb (99%), spinosad (97%), and coumaphos (75%), whereas azinphos-methyl (59%), chlorpyrifos (54%), and carbofuran (43%) were partially removed (Figure 4). When comparing the insecticide removal of commercial laccase with the BS-derived crude enzyme extract, a significant difference (*p* < 0.05) was observed with only 2 out of the 10 (azinphos-methyl and carbofuran) insecticides tested.

Pesticides such as atrazine, metolachlor, coumaphos, azinphos-methyl, and kresoxim-methyl that were almost completely or partially removed all possess one or more substituted amine aromatic rings, which would effectively lower their redox potential, thereby making them the substrate of choice for oxidation by laccase. Also, several pesticides considered in the study have a similar chemical composition and structural resemblance to well-known laccase mediators and catalytic enhancers. Such compounds possess nitrogen-bearing functional groups as substitutions that impart an overall negative charge to the molecule, making it favorable for transformation by laccase (Tran et al., 2010; Margot et al., 2013).

Removal of chlorpyrifos obtained during this study was relatively low when compared to the laccase extracted from *Pseudomonas* sp. (80.56%) grown over agro-waste (Chauhan and Jha, 2018) and commercial laccase from *T. versicolor* (90%) with the presence of mediators tested in a buffer solution (Jin et al., 2016). On the contrary, other insecticides (carbofuran, acetamiprid, parathion, malathion, and chlorfenvinphos) that were tested for the BS-derived crude enzyme extract, and acetamiprid, parathion, malathion, and chlorfenvinphos, which were tested for commercial laccase, remained recalcitrant during the 24 h experiment.

In the case of the BS-derived and commercial laccase treated insecticide cocktail, a similar trend was observed as in the herbicide cocktail, where compounds that had been removed to a lesser extent, when treated separately, were found to be removed to a greater extent when treated as a cocktail of related compounds. In the BS-derived crude enzyme extract insecticide mixture, elevated removal was achieved for coumaphos (91%), azinphos-methyl (90%), and chlorpyrifos (83%), which were 8, 33 and 22% higher, respectively, than when they were treated separately (Figure 5). Although mediocre yet the removal of aldicarb (60%) and spinosad (52%) decreased by 39 and 46% respectively, when treated as a cocktail. When comparing the insecticide removal with BS-derived crude enzyme extract for cocktail and isolated experiments, a significant difference (*p* < 0.05) was observed for 3 out of 10 insecticides tested (azinphos-methyl, aldicarb, and spinosad). With the commercial laccase, the removal improved by 12, 19, and 14% for coumaphos, azinphos-methyl, and chlorpyrifos, respectively, in the insecticide cocktail compared with these insecticides treated separately (Figure 5). Carbofuran (63%) removal in the insecticide cocktail was similar to that reported in studies done by Kupski et al. (2019) in the presence of a mediator system. However, the removal of aldicarb and sinosad neither increased nor decreased after 24 h.

#### 3.3.3 Removal of Fungicides

In isolated BS-derived crude enzyme extract treatments, kresoxim-methyl (96%) and pyraclostrobin (91%) exhibited near-complete removal, while carbendazim (59%) and fludioxonil (56%) underwent partial degradation, followed by pyrimethanil (29%) with the lowest degradation (Figure 4). With the commercial laccase, five of the fungicides tested (kresoxim-methyl, pyraclostrobin, carbendazim, thiabendazole, and iprodione) had a more than 90% removal rate. The partial and lowest degradation were obtained with fludioxonil (51%) and pyrimethanil (18%) (Figure 4). The removal of pyrimethanil was inconsistent with the study done by Jin et al. (2016), where they reported a 100% removal of pyrimethanil in 24 h of degradation with commercial laccase from *T. versicolor* (Jin et al., 2016)*.* When comparing the fungicide removal of commercial laccase with BS-derived crude enzyme extract, a significant difference (*p* < 0.05) was observed with only two out of nine (thiabendazole and iprodione) fungicides tested.

In the fungicide cocktail treatment, kresoxim-methyl (99%) once again exhibited near-complete removal with both laccases. Enhanced removal of fungicides by BS-derived crude enzyme extract was also achieved for carbendazim (86%) and fludioxonil (81%). However, significant differences (*p* < 0.05) were only observed for two out of nine fungicides tested (fludioxonil and pyrimethanil) while comparing the fungicide removal with BS-derived crude enzyme extract for the cocktail and isolated experiments. During the cocktail experiment with commercial laccase, the removal percentages of fludioxonil and boscalid, which had been recalcitrant during the isolated experiment, increased to 69 and 21%, respectively (Figure 5). The removal of pesticides that were initially recalcitrant to oxidation by laccase when treated separately but which underwent removal in the cocktail occurs *via* a free radical of oxygen localized within the aromatic ring structures, followed by the polymerization of radicals to form oligomers that eventually fall out of solution by precipitation or undergo subsequent breakdown of the labile polyaromatic molecule (Ba et al., 2014). When subjected to enzymatic treatment as a mixture of compounds, the radicals generated undergo a similar hetero-polymerization *via* cross-coupling reactions. The binding of existing radicals to recalcitrant pesticides makes them more favorable for oxidative degradation by laccase. Similar effects have been explained by Touahar et al. (2014) and Kumar and Cabana (2016) when they studied the removal of pharmaceuticals in a cocktail using laccase. Also, the phenolic ring structure has an inherent laccase mediator effect, which drives the oxidation of recalcitrant substrates (d’Acunzo and Galli, 2003). Pyrimethanil and trifloxystrobin did not undergo any transformation in the mixture either by commercial laccase or BS-derived enzyme extract. Table 3 summarizes the previous findings for the removal of pesticides with free laccases.

**TABLE 3:** Previous findings for removal of pesticides with free laccases.

Compound	Enzyme	Mediator	Time (h)	% Removal	References
Glyphosate	Laccase	ABTS	24	40.9	Pizzul et al. (2009)
Dymron	Laccase	ABTS	24	90	(Maruyama et al., 2006)
Chlorpyrifos	Laccase	—	48	70	(Wang et al., 2016)
Chlorpyrifos	Laccase	—	15	29	Rudakiya et al. (2020)
Trifluralin	Laccase	Guaiacol	24	100	(Bansal et al., 2018)
Chlorpyrifos	Laccase	Vanillin	192	90	Jin et al. (2016)
Chlorothalonil	Laccase	Acetosyringone	192	90	
Isoproturon	Laccase	Violuric acid	24	100	
Atrazine	Laccase	HBT	192	90	
Pyrimethanil	Laccase	Violuric acid	24	100	
Chlorpyrifos	Laccase	Vanillin	48	100	(Xie et al., 2013)
Bentazon	Laccase	Vanillin	3	54	Kupski et al. (2019)
Carbofuran	Laccase	Vanillin	3	39	
Diuron	Laccase	Vanillin	3	46	
Pyraclostrobin	Laccase	Vanillin	3	78	

This experiment is the first of its kind in which free laccase has been used to study the removal of a broad range of pesticides without the use of any extraneous mediators or fungal species for bioremediation purposes. Another related study based on pesticide degradation for a period of 6 days employing manganese peroxidase (MnP) for the detoxification of 22 compounds was published by Pizzul et al. (2009) in which the action of MnP in the presence of MnSO_4_ and Tween-80 detergent improved the degradation rate of tested pesticides (Pizzul et al., 2009). Comparing the results attained in this study during 24 h of treatment, without the addition of any mediators, with the results conducted by Pizzul et al. (2009), it would be safe to state that laccase is a viable candidate for enzymatic remediation of the studied pesticides.

## Conclusion

This study investigated the use of biosolids for laccase production by inoculating *P. dryinus*. Among the four different types of BS tested, sterilized BS was found to be the most efficient one by producing a maximum laccase activity of around 163 U/g of dry substrate along with other ligninolytic enzymes. Crude enzyme extract containing laccase produced from SBS bioaugmented *P. dryinus* establishes the broad range specificity of *P. dryinus* toward pesticides and their effective elimination, emphasizing its potential as a bioremediation agent without the use of any additional mediators. The catalytic effect in a mixture of related compounds was similar to the laccase mediator effect. This highlights the use of laccase as an essential green chemistry tool for wastewater remediation, facilitating the elimination of multiple pollutants from municipal wastes, hospital wastes, and agricultural run-offs. Further work would be focused on increasing the catalytic potential and facilitating the recovery of enzymes from a reaction mixture. This calls for exploring suitable supports for laccase immobilization with high activity recovery and recyclability of the immobilized enzyme.

## Data Availability

The raw data supporting the conclusion of this article will be made available by the authors, without undue reservation.
